# Low-Cytotoxic Synthetic Bromorutaecarpine Exhibits Anti-Inflammation and Activation of Transient Receptor Potential Vanilloid Type 1 Activities

**DOI:** 10.1155/2013/795095

**Published:** 2013-11-28

**Authors:** Chi-Ming Lee, Jiun-An Gu, Tin-Gan Rau, Che-Hsiung Yang, Wei-Chi Yang, Shih-Hao Huang, Feng-Yen Lin, Chun-Mao Lin, Sheng-Tung Huang

**Affiliations:** ^1^Graduate Institute of Medical Sciences, College of Medicine, Taipei Medical University, No. 250, Wu-xing Street, Taipei 110, Taiwan; ^2^Institute of Chemical Engineering, College of Engineering, National Taipei University of Technology, Taipei, Taiwan; ^3^Department of Food and Beverage Management, Taipei College of Maritime Technology, Taipei, Taiwan; ^4^Department of Internal Medicine, School of Medicine, Taipei Medical University, No. 250, Wu-xing Street, Taipei 110, Taiwan; ^5^Department of Biochemistry, College of Medicine, Taipei Medical University, No. 250, Wu-xing Street, Taipei 110, Taiwan; ^6^Orthopedics Research Center, Taipei Medical University Hospital, Taipei, Taiwan; ^7^Institute of Biochemical and Biomedical Engineering, College of Engineering, National Taipei University of Technology, Taipei, Taiwan

## Abstract

Rutaecarpine (RUT), the major bioactive ingredient isolated from the Chinese herb *Evodia rutaecarpa*, possesses a wide spectrum of biological activities, including anti-inflammation and preventing cardiovascular diseases. However, its high cytotoxicity hampers pharmaceutical development. We designed and synthesized a derivative of RUT, bromo-dimethoxyrutaecarpine (Br-RUT), which showed no cytotoxicity at 20 **μ**M. Br-RUT suppressed nitric oxide (NO) production and tumor necrosis factor-**α** release in concentration-dependent (0*~*20 **μ**M) manners in lipopolysaccharide (LPS)-treated RAW 264.7 macrophages; protein levels of inducible NO synthase (iNOS) and cyclooxygenase-2 induced by LPS were downregulated. Br-RUT inhibited cell migration and invasion of ovarian carcinoma A2780 cells with 0*~*48 h of treatment. Furthermore, Br-RUT enhanced the expression of transient receptor potential vanilloid type 1 and activated endothelial NOS in human aortic endothelial cells. These results suggest that the synthetic Br-RUT possesses very low cytotoxicity but retains its activities against inflammation and vasodilation that could be beneficial for cardiovascular disease therapeutics.

## 1. Introduction

Evodiamine (EVO) and rutaecarpine (RUT), major bioactive ingredients isolated from the Chinese herb *Evodia rutaecarpa* [1], possess a wide spectrum of biological activities [2]. Inflammation and low oxygen diffusion are characteristics of atherosclerosis. EVO repressed cyclooxygenase (COX)-2 and inducible nitric oxide (NO) synthase (iNOS) expression mediated via inhibition of hypoxia-inducible factor (HIF)-1*α* under hypoxic conditions [3]. Therefore, EVO is considered an effective therapeutic agent against inflammatory diseases involving hypoxia. Vasorelaxant effects of EVO and RUT in rat isolated mesenteric arteries were reported to be associated with Ca^2+^ flux activity [4, 5]. RUT lowered blood pressure through the endothelial Ca^2+^-NO-cGMP pathway to reduce smooth muscle tone [6].

The calcitonin gene-related peptide (CGRP), a major neurotransmitter of capsaicin-sensitive sensory nerves, plays a key role in maintaining endothelial homoeostasis. Decreased plasma CGRP levels caused cardiac susceptibility to ischemia-reperfusion injury, and antihypertensive therapy with RUT reversed cardiac susceptibility to reperfusion injury by stimulating CGRP release [7, 8]. The CGRP can counteract angiotensin (Ang) II-induced endothelial progenitor cell senescence through downregulating NADPH oxidase and reactive oxygen species (ROS) production [9]. Stimulation of endogenous CGRP release via activation of vanilloid receptors plays an important role in the vasodilatory effects of RUT [10, 11]. Activation of transient receptor potential vanilloid type 1 (TRPV1), a ligand-gated cationic channel, by EVO in endothelial cells may protect against certain cardiovascular diseases (CVDs) such as hypertension and stroke [12, 13]. Okada et al. reported that TRPV1 is a potential drug target for improving the outcome of inflammatory fibrosis [14]. NO release with consequent activation of endothelial (e)NOS confers vascular relaxation mediated by CGRP and TRPV1 stimulation [15]. The effect of EVO in TRPV1-dependent atheroprotection was further confirmed in mice [16]. Sheu et al. reported that RUT is a potential therapeutic agent for arterial thromboses because of its in vivo antiplatelet effect [17, 18]. Alkaloid compounds also exhibit anticancer activities both in vitro and in vivo by inducing cell-cycle arrest or apoptosis [19].

RUT and EVO showed quite high toxicity to porcine brain capillary endothelial cells (ECs) [20], which limits their application in vascular diseases. A variety of structural modifications of natural products were designed and synthesized for superior biological applications. Structure-activity relationship studies were further performed and are in progress [21–23]. RUT analogues were designed and synthesized to activate TRPV1 for enhanced vasodilator and hypotensive effects. The 14-N atom of RUT is critical for its activity [24]. Synthetic derivatives of RUT in this study exhibited very low cytotoxicity, but they still maintained their anti-inflammatory activity and TRPV1-upregulating effects. Results provide insights into the use of this TRPV1 agonist from RUT in vascular disease therapeutics.

## 2. Materials and Methods

### 2.1. Chemicals and General Methods

All chemicals were purchased from Acros Organics (Geel, Belgium), Sigma-Aldrich (St. Louis, MO), Showa Chemical Industry (Tokyo, Japan), or TCI America (Portland, OR) and were used without further purification. All reactions requiring anhydrous conditions were performed in oven-dried glassware under an Ar or N_2_ atmosphere. Chemicals and solvents were either used without purification or purified by standard techniques. Analytical thin-layer chromatography (TLC) was performed on glass plate-mounted silica gel 60F254 (Merck) at a thickness of 0.2 mm. Flash column chromatography was performed using Silicycle silica gel 60. Synthesized compounds were characterized using 1H nuclear magnetic resonance (NMR) (Bruker Avance 500 MHz, Billerica, MA) and Fourier-transformed infrared spectroscopy (FT-IR).

### 2.2. Synthesis of Bromo-(Br-)RUT Derivatives

**Scheme 1:**
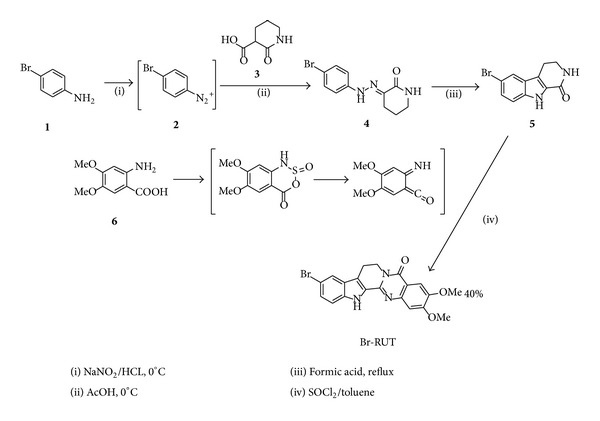
Synthesis of bromo-dimethoxyrutaecarpine (Br-RUT).

2-Amino-4,5-dimethoxybenzoic acid (6 of Scheme 1) (0.4 g, 2 mmol) was dissolved in toluene (6 mL) that had been cooled to 0°C. Thionyl chloride (0.75 mL, 10 mmol) was added dropwise to the ice-cold solution. The reaction mixture was heated to 70~80°C and stirred for 1 h. The solution was heated to reflux for 10 min, was allowed to cool to 23°C, and was concentrated under reduced pressure. The resulting residue was redissolved in toluene (6 mL). A compound of 2,3-piperidinedione-3-(4-bromophenyl) hydrazone (5 of Scheme 1) (0.35 g, 1.37 mmol) was added to the solution. The reaction mixture was heated to reflux and stirred overnight. The solution was concentrated on a rotary evaporator, 10% sodium carbonate aqueous was added (200 mL), and the reaction was extracted with dichloromethane (3 × 200 mL). The organic layer was dried over anhydrous MgSO_4_, the solids were filtered through a fritted Büchner funnel, and the solution was concentrated under reduced pressure. The residue was purified by column chromatography (elution with DCM : methanol of 40 : 1), affording Br-RUT as a solid.

### 2.3. Cell Culture

The RAW 264.7 macrophage cell line and A2780 ovarian carcinoma cells were grown in Dulbecco's modified Eagle's medium (DMEM) containing 10% fetal bovine serum (FBS), 100 U/mL penicillin, 100 *μ*g/mL streptomycin, 4 mM L-glutamine, 4.5 g/L glucose, 1 mM sodium pyruvate, and 1.5 g/L sodium bicarbonate at 37°C in a humidified atmosphere with 5% CO_2_. Primary human aortic ECs (HAECs) and human coronary artery ECs (HCAECs) were grown in a MesoEndo Endothelial Cell Growth Medium Kit (Cell Applications, San Diego, CA) supplemented with 10% FBS at 37°C in a humidified atmosphere with 5% CO_2_.

### 2.4. Nitrite Assay

NO production in cell culture supernatant was evaluated by measuring the nitrite concentration. The nitrite concentration was detected with the Griess reaction. RAW 264.7 macrophages were plated at a density of 2 × 10^5^ cells/mL in 24-well plates for 24 h, followed by cotreatment with different concentrations of Br-RUT and lipopolysaccharide (LPS) (100 ng/mL) for 24 h. The amount of nitrite in the samples was detected using the Griess reagent (1% sulfanilamide in 5% phosphoric acid and 0.1% naphthyl ethylenediamine dihydrochloride dihydrochloride in water). Data are reported as the mean ± standard error of the mean (SEM) of three independent determinations [25].

### 2.5. TNF-*α* Assay

Soluble cytokines were tested in supernatants of cultured RAW 264.7 macrophages by an enzyme-linked immunosorbent assay (ELISA). RAW 264.7 macrophages were plated at a density of 10^4^ cells/mL in 96-well plates for 12 h, followed by treatment with different concentrations of Br-RUT for 1 h, then treatment with LPS (100 ng/mL) for 12 h. TNF-*α* in cell supernatants was detected using a mouse TNF-*α* Quantikine kit (R&D Systems, Minneapolis, MN) which was carried out according to the manufacturer's instructions. The absorbance was read at 450 nm with an ELISA plate reader. Data are reported as the mean ± SEM of three independent determinations.

### 2.6. Western Blot Analysis

Protein samples were resolved by sodium dodecylsulfate polyacrylamide gel electrophoresis (SDS-PAGE) and electrotransferred onto a polyvinylidene difluoride membrane. The membrane was incubated with a primary antibody at 4°C overnight and then incubated with a horseradish peroxidase (HRP)-conjugated secondary immunoglobulin G antibody; immunoreactive bands were visualized with PerkinElmer enhanced chemiluminescent reagents [26].

### 2.7. Cell Viability Assay

An MTT assay to test the cytotoxicity of reagents and cell viability was performed as described previously, based on conversion of the yellow tetrazolium salt to the purple formazan product [27]. Cells (10^4^ cells/well) were grown on a 96-well plate supplemented with culture medium. Cells were treated with Br-RUT (0~20 *μ*M) for 24 h, and an MTT stock solution (5 mg of MTT/mL of phosphate-buffered saline; PBS) was added to the growing cultures for 2 h. The absorbance was measured with a spectrophotometer at 560 nm. A blank with DMSO alone was measured as a reading control.

### 2.8. Wound Healing Migration Assay

A2780 cells at a density of 2 × 10^5^ cells/well in 6-well plates were incubated at 37°C for 24 h. Cells were scratched with a 200-*μ*L pipette tip, and cells in the plate were washed with PBS, and then new complete medium was added and treated with or without 1 and 2.5 *μ*M of cantharidin for 24 h; 0.5% DMSO served as a vehicle control. At the end of incubation, cells were examined and photographed under a fluorescence microscope. The number of cells that had migrated into the scratched area was calculated as described elsewhere [28].

### 2.9. In Vitro Invasion Assay

A2780 cell migration or invasion was evaluated using 24-well Transwell inserts (8 *μ*m pore filters, Merck Millipore) individually coated with Matrigel (BD Biosciences, Bedford, MA). A2780 cells (2 × 10^4^ cells/well) were cultured for 24 h in serum-free DMEM, and then cells were placed in the upper chamber of the Transwell insert and treated with 0.5% DMSO (as a control) or Br-RUT (1.25 or 2.5 *μ*M) for 24 h. Medium containing 10% FBS was placed in the lower chamber. At the end of incubation, nonmigrated cells were removed using a cotton swab; invaded cells maintained in the upper chamber were fixed with 4% formaldehyde and stained with 2% crystal violet. On the lower surface of the filter, cells that had penetrated were counted and photographed under a phase-contract microscope at 200x magnification. Three independent experiments were performed as described elsewhere [28].

## 3. Results

### 3.1. Design and Synthesis of Br-RUT

The synthetic schemes for preparing Br-RUT derivatives are outlined in Scheme 1. The main intermediate, substituted carboline (5), was prepared from substituted aniline following a procedure described by Henecka et al. [29] and Narayanan et al. [30]. The aniline (1) underwent the Sandmeyer reaction to give a diazonium salt, which was then coupled to carboxylic acid (3) to yield hydrazone (4), and treatment of hydrazone (4) in an acidic condition gave carboline (5) in a 56% yield (3 steps). Carboline (5) (see Figure S1 in Supplementary Material available online at http://dx/doi.org/10.1155/2013/795095) was then coupled with an *in situ* activated substituted o-aminobenzoic acid derivative (6), which was pretreated with thionyl chloride in the presence of toluene at 70~80°C to provide Br-RUT in a 40% overall yield (4 steps). The chemical structures of the synthetic intermediates and final products were determined by ^1^H and ^13^C NMR, IR, and mass spectrometry. FT-IR (KBr, cm-1): 3330 (N–H) and 1668 (carbonyl group) (Supplementary Figure S2). 1H-NMR (D-DMSO, ppm) (Supplementary Figure S2): *δ*3.13 (*t*, *J* = 6.8 Hz, 2H), 3.87 (s, 3H), 3.91 (s, 3H), 4.41 (*t*, *J* = 6.8 Hz, 2H), 7.05 (s, 1H), 7.34 (*dd*, *J* = 8.6 1.4, 1H), 7.41 (*d*, *J* = 8.6, 1H), 7.47 (*s*, 1H), 7.85 (*s*, 1H), 11.97 (*s*, 1H) (Supplementary Figure S3). MS-EI (*m*/*z*): calcd. 426.2; found 425.6 (Supplementary Figure S4).

### 3.2. NO and TNF-*α* Release by LPS-Treated Macrophages was Suppressed by Br-RUT

**Figure 1:**
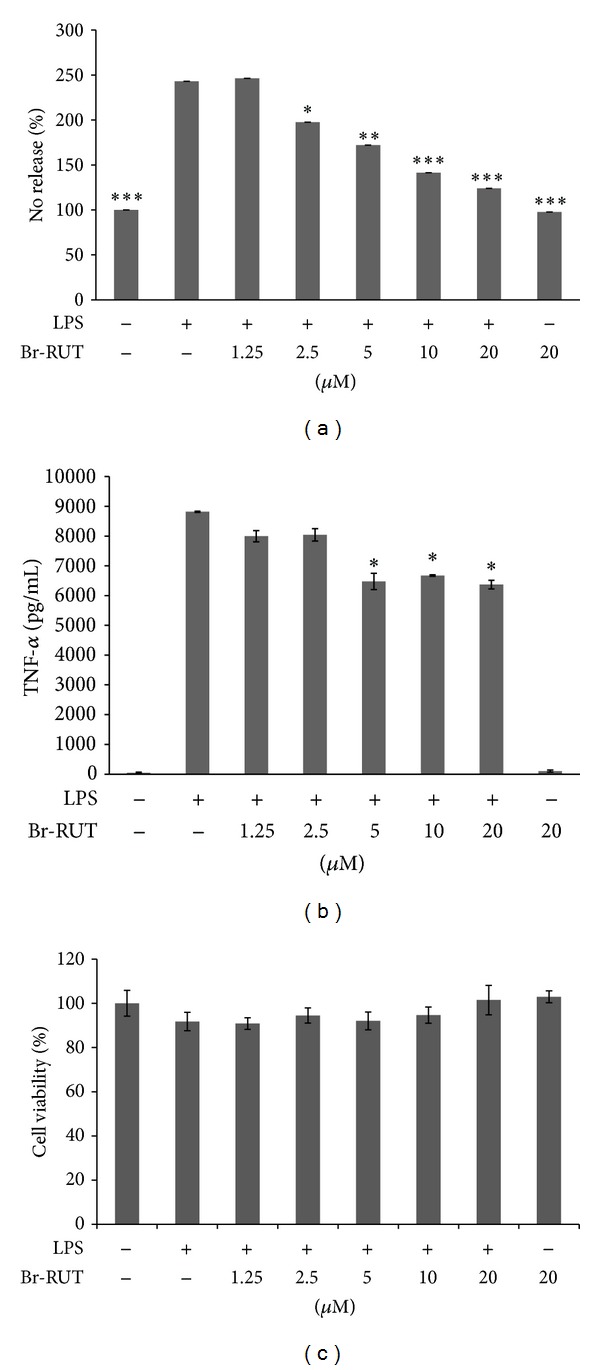
Effects of bromo-dimethoxyrutaecarpine (Br-RUT) on nitric oxide (NO) and tumor necrosis factor (TNF)-*α* releases by lipopolysaccharide (LPS)-treated (100 ng/mL) RAW 264.7 macrophages. (a) NO levels were detected in culture medium using the Griess reaction. The percentage of untreated cells was set as the control to 100%. (b) TNF-*α* release in cell supernatants was detected using a mouse TNF-*α* Quantikine kit. (c) Cell viability upon Br-BUT treatment for 24 h in an MTT assay. Statistical significance is indicated compared to LPS treatment. (*P < 0.05, **P < 0.001, ***P < 0.001).

NO production by LPS-treated RAW264.7 macrophages increased compared to untreated cells. Pretreatment for 1 h with synthetic Br-RUT suppressed NO production in a concentration-dependent (0~20 *μ*M) manner (*P* < 0.01) (Figure 1(a)). TNF-*α* released into the medium also showed consistent potency in a concentration-dependent (0~20 *μ*M) (Figure 1(b)) suppressive effect. Suppressive effects were not due to the reduction of cell number because Br-RUT showed no cytotoxicity at concentrations of 0~20 *μ*M (Figure 1(c)).

### 3.3. Br-RUT Suppressed iNOS and COX-2 Protein Expressions by LPS-Treated Macrophages

**Figure 2:**
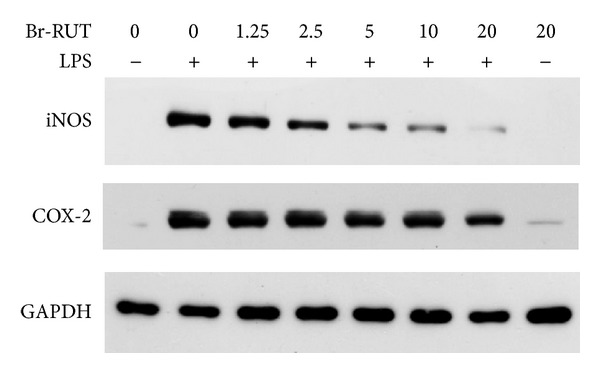


We investigated the effects of Br-RUT on protein levels of iNOS and COX-2. LPS-treated RAW 264.7 macrophages exhibited significantly elevated protein amounts of iNOS and COX-2, while Br-RUT suppressed their expressions in concentration-dependent manners (Figure 2). GAPDH protein levels of the loading controls remained constant.

### 3.4. Br-RUT Inhibited Cell Migration/Invasion

**Figure 3:**
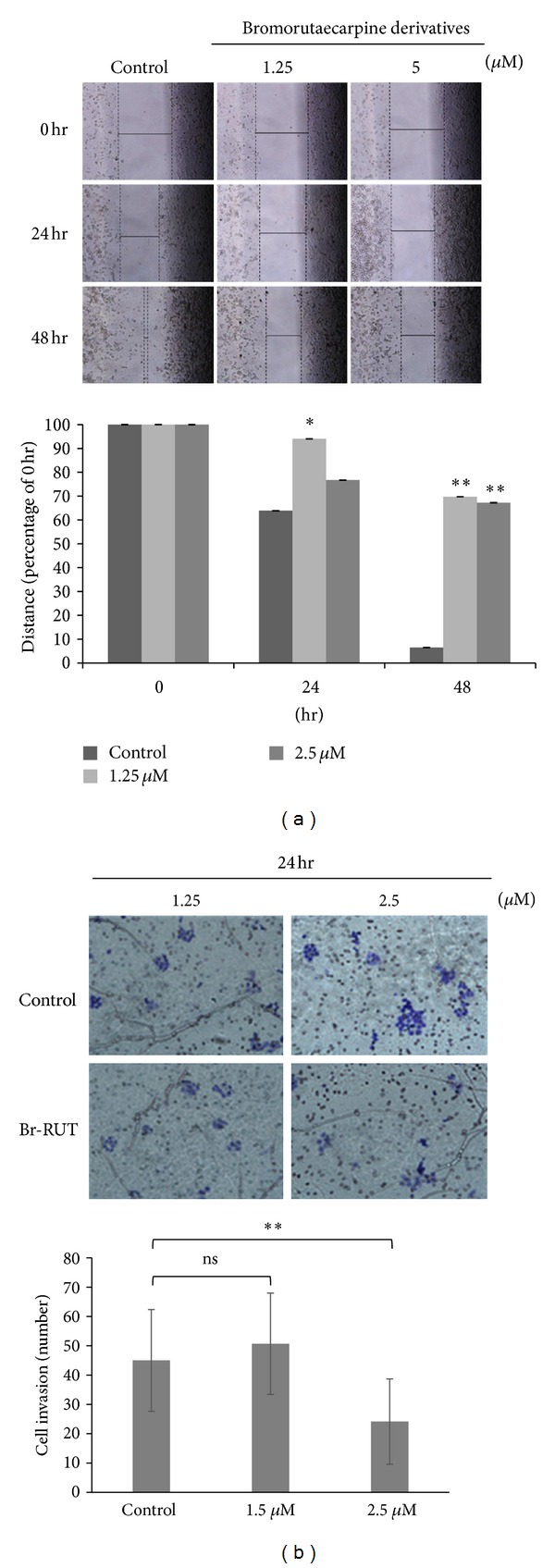
Effects of bromo-dimethoxyrutaecarpine (Br-RUT) on cell migration and invasion. Cell migration (a) and invasion (b) were detected following Br-RUT treatment for 0~48 h and photographed with a microscope (upper panel). Stained cells were counted and calculated in three random regions for each sample, results are presented as the mean ± SD from triplicate experiments, and the statistical analysis is shown in the lower panel.

The effects of Br-RUT on inhibiting cell migration and invasion were investigated. As illustrated in Figure 3(a), wound healing assays used an ovarian carcinoma A2780 cell line in the presence of Br-RUT (0~5 *μ*M) for 0~48 h. The migration velocity was measured using imaging software, and Student's *t*-test was used for the statistical analysis. Br-RUT showed significant effects against cell migration. Br-RUT (0~2.5 *μ*M) treatments for 24 h also exhibited invasion-inhibitory activity in a transwell assay (Figure 3(b)).

### 3.5. Br-RUT Activated TRPV1 and eNOS

**Figure 4:**
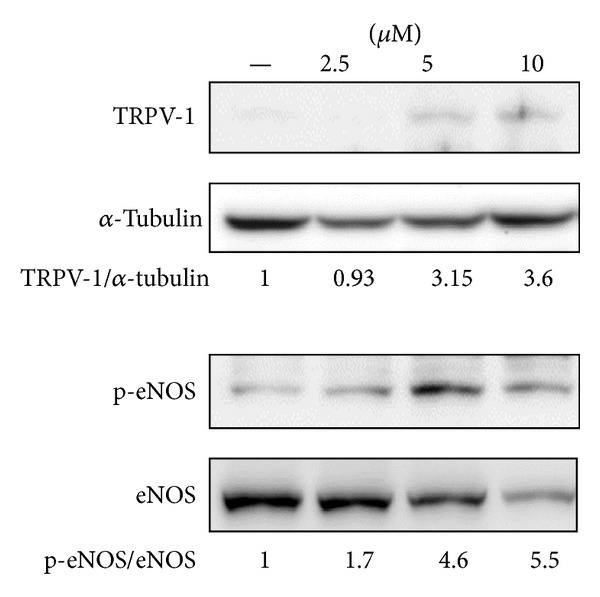
Effects of bromo-dimethoxyrutaecarpine (Br-RUT) on transient receptor potential vanilloid type 1 (TRPV1) expression and endothelial nitric oxide synthase (eNOS) phosphorylation in human aortic endothelial cells (HAECs). The densitometric ratio is indicated.

TRPV1 is reportedly present in ECs of arteries. To validate the expression of TRPV1 in the endothelium, the TRPV1 protein of HAECs was detected by immunoblotting. Br-RUT treatment (10 *μ*M) for 24 h increased TRPV1 protein amounts by 3.6-fold compared to the control group after normalization with *α*-tubulin levels (Figure 4). We further examined the effect of Br-RUT on the phosphorylation of NOS in HAECs because NO production is mainly regulated by eNOS phosphorylation. Br-RUT treatment (10 *μ*M) for 24 h significantly increased eNOS phosphorylation by 5.5-fold compared to the control group after normalization with unphosphorylated eNOS (lower panel). Similar results were found in HCAECs (Supplementary Figure S5). Br-RUT upregulated the expression of TRPV1 and activated eNOS of ECs.

## 4. Discussion

The anti-inflammatory activities of EVO and RUT were reported [4, 31], but they act through different mechanisms, because EVO inhibits COX-2 induction and nuclear factor (NF)-*κ*B activation in LPS-treated RAW 264.7 cells, while RUT does not [32]. RUT was reported to cause vasodilator effects by stimulating CGRP synthesis and release via activation of TRPV1. Its analogues were designed and synthesized for better vasodilator effects. Structural modifications of RUT and EVO were designed to enhance their biological activities. However, increased cytotoxicity hampers their application to vascular disorders [20, 33]. Br-RUT, a novel analogue synthesized in this study, had very low cytotoxicity and showed anti-inflammatory and migration/invasion-suppression activities that were beneficial with reduced side effects, so it has the potential to be developed for pharmaceutical applications.

RUT exerts different mechanisms to modulate signaling pathways, which resulted in different cell fates with diverse cell types and growth conditions [34]. RUT relaxed vascular smooth muscles by stimulating CGRP release via activation of vanilloid receptor subtype 1 [35]. It also antagonized Ang II-induced decreases in cellular NO contents and eNOS activities in rat vascular smooth muscle cells [36]. Compared to RUT, Br-RUT showed much less cytotoxicity, but it retained the anti-inflammatory activity. Br-RUT suppressed iNOS in macrophages, while it activated eNOS in ECs. Results showed that Br-Rut, derived from RUT, has enhanced beneficial effects and reduced adverse activities. Br-RUT, a modified derivative of RUT, retains the activities of improving cardiac and vasodilation functions but has fewer side effects.

Cells of the heart arise through a series of epithelial-to-mesenchymal transitions (EMTs), followed by formation of new epithelial structures by the reverse process of a mesenchymal-to-epithelial transition (MET) and then differentiation into cardiomyocyte and endocardial lineages. ECs from the atrioventricular canal undergo a tertiary EMT, called EndMT (due to the original properties of the tissue), and cells later assemble into the atrioventricular valvuloseptal complex [37]. Recent studies revealed that many cardiac diseases caused by inflammation are associated with fibrosis in the heart. Fibrosis is characterized by the accumulation of fibroblasts and the production of extracellular matrix. EndMT plays important roles in cardiac fibroblast formation during pathological progression. EndMT is regulated by signaling pathways mediated by inflammation-associated cytokines [38]. The endothelium is a promising target for drug treatment because it is in direct contact with the bloodstream. Br-RUT possesses anti-inflammatory activity and suppresses migration/invasion activities, phenomena that are associated with EndMT-associated fibrosis. Activation of TRPV1 and eNOS by Br-RUT suggests its potential for preventing vasorelaxation/hypertension. We therefore propose that inhibition of mechanisms for the formation of cardiac fibroblasts via EndMT by Br-RUT may provide a new strategy for heart disease therapeutics.

## 5. Conclusions

Br-RUT, a modified derivative of RUT, possesses very low cytotoxicity but retains its activity against inflammation. Treatment with Br-RUT enhanced TRPV1 and eNOS activities that may be beneficial by improving cardiac and vasodilation function.

## Supplementary Material

Br-RUT was Designed and synthesized according to the Scheme 1. Fig. S1 is the FT-IR spectrum of Br-RUT alone with its chemical structure as shown in the insert (Left). The two signature IR bands of 1668 cm^−1^and 3330 cm^−1^ reflect the corresponding functional groups, the carbonyl and the N-H stretching, respectfully. Fig. S2 is the ^1^H NMR spectrum of Br-RUT in D_6_-DMSO alone with its chemical structure as shown in the insert. The protons of the chemical structure has been assigned with alphabetical letters (a through i), and each letter corresponds to the assignments of the peaks within spectrum.
Fig. S3 is the effects of Br-RUT on TRPV-1 expression and eNOS phosphorylation in human coronary artery endothelial cells (HCAECs). To validate the expression of TRPV1 in the endothelium, the TRPV1 protein of HCAECs was detected by immunoblotting. Br-RUT treatment (0-10 *μ*M) for 24 h increased TRPV1 protein amounts compared to the control group after normalization with *α*-tubulin levels. Br-RUT treatment (0-10 *μ*M) for 24 h increased eNOS phosphorylation compared to the unphosphorylated eNOS (lower panel).
