# Supplementary material for: Low-Cytotoxic Synthetic Bromorutaecarpine Exhibits Anti-Inflammation and Activation of Transient Receptor Potential Vanilloid Type 1 Activities
Source: Biomed Res Int. 2013 Nov 28;2013:795095. doi: 10.1155/2013/795095 (PMC3863474; doi:10.1155/2013/795095)
Supplement: Supplementary file 1 — Br-RUT was Designed and synthesized according to the Scheme 1. Fig. S1 is the FT-IR spectrum of Br-RUT alone with its chemical structure as shown in the insert (Left). The two signature IR bands of 1668 cm−1and 3330 cm−1 reflect the corresponding functional groups, the carbonyl and the N-H stretching, respectfully. Fig. S2 is the 1H NMR spectrum of Br-RUT in D6-DMSO alone with its chemical structure as shown in the insert. The protons of the chemical structure has been assigned with alphabetical letters (a through i), and each letter corresponds to the assignments of the peaks within spectrum. Fig. S3 is the effects of Br-RUT on TRPV-1 expression and eNOS phosphorylation in human coronary artery endothelial cells (HCAECs). To validate the expression of TRPV1 in the endothelium, the TRPV1 protein of HCAECs was detected by immunoblotting. Br-RUT treatment (0-10 μM) for 24 h increased TRPV1 protein amounts compared to the control group after normalization with α-tubulin levels. Br-RUT treatment (0-10 μM) for 24 h increased eNOS phosphorylation compared to the unphosphorylated eNOS (lower panel). [file 795095.f1.pdf]

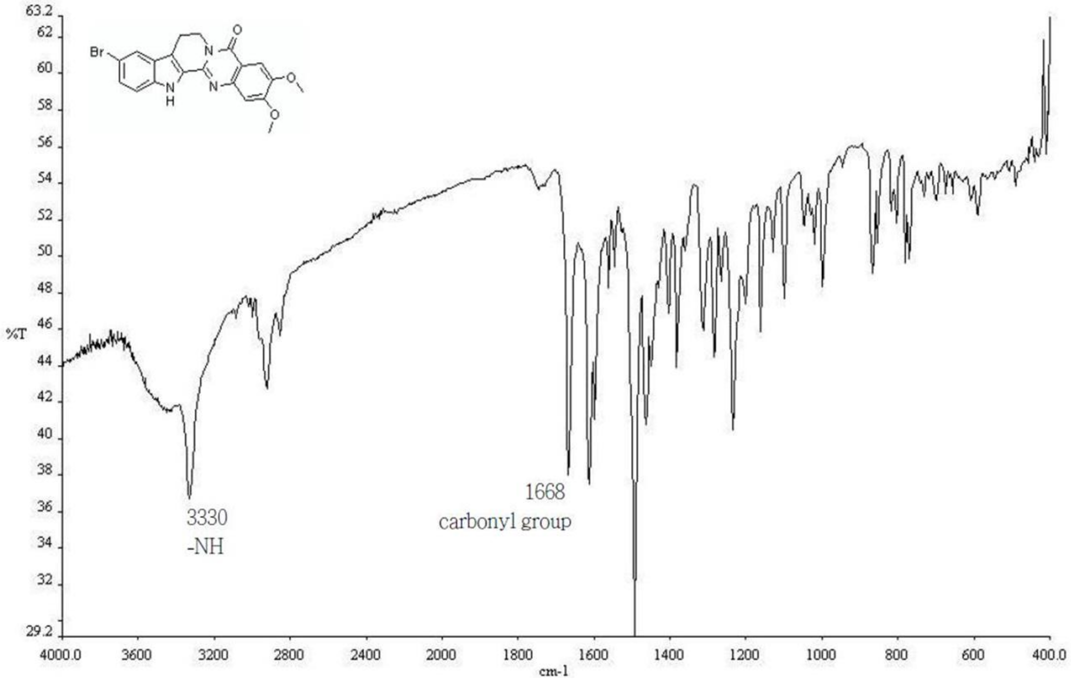

Supplementary Fig. S1: FT-IR (KBr, cm-1) spectrum of Br-RUT

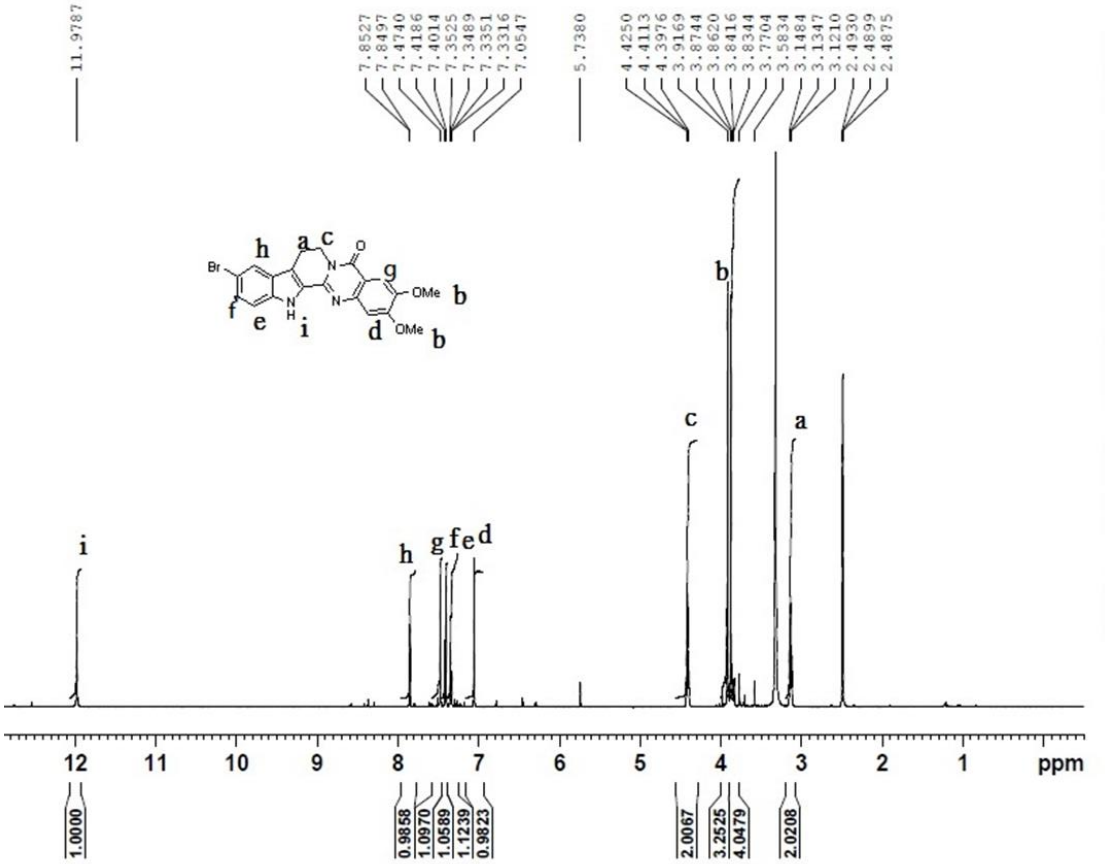

Supplementary Fig. S2: 1H NMR spectrum of Br-RUT

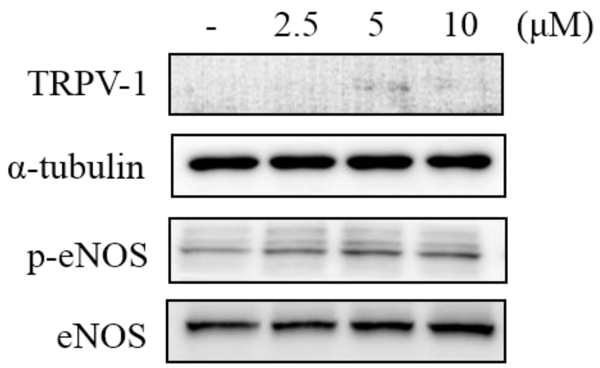

Supplementary Fig. S3: Effects of Br-RUT on TRPV-1 expression and eNOS phosphorylation in human coronary artery endothelial cells (HCAECs)
